# The prevalence of nonlinearity and detection of ecological breakpoints across a land use gradient in streams

**DOI:** 10.1038/s41598-019-40349-4

**Published:** 2019-03-07

**Authors:** Sarah C. D’Amario, Daniel C. Rearick, Christina Fasching, Steven W. Kembel, Emily Porter-Goff, Daniel E. Spooner, Clayton J. Williams, Henry F. Wilson, Marguerite A. Xenopoulos

**Affiliations:** 1 Department of Biology, Trent University, Peterborough, ON Canada; 2 Environmental and Life Sciences Graduate Program, Trent University, Peterborough, ON Canada; 3 Département des sciences biologiques, Université du Québec à Montréal, Montréal, QC Canada; 4 Department of Biology, Lock Haven University, Lock Haven, PA USA; 5 Rubenstein School of Environment and Natural Resources, University of Vermont, Burlington, VT USA; 6 Agriculture and Agri-Food Canada, Brandon, MB Canada

## Abstract

Human activities can alter aquatic ecosystems through the input of nutrients and carbon, but there is increasing evidence that these pressures induce nonlinear ecological responses. Nonlinear relationships can contain breakpoints where there is an unexpected change in an ecological response to an environmental driver, which may result in ecological regime shifts. We investigated the occurrence of nonlinearity and breakpoints in relationships between total dissolved nitrogen (TDN), total dissolved phosphorus (TDP), and total dissolved carbon (DOC) concentrations and ecological responses in streams with varying land uses. We calculated breakpoints using piecewise regression, two dimensional Kolmogorov-Smirnov (2DKS), and significant zero crossings (SiZer) methods. We found nonlinearity was common, occurring in half of all analyses, with some evidence of multiple breakpoints. Linearity, by contrast, occurred in less than 14% of cases, on average. Breakpoints were related to land use gradients, with 34–43% agricultural cover associated with DOC and TDN breakpoints, and 15% wetland and 9.5% urban land associated with DOC and nutrient breakpoints, respectively. While these breakpoints are likely specific to our study area, our study contributes to the growing literature of the prevalence and location of ecological breakpoints in streams, providing watershed managers potential criteria for catchment land use thresholds.

## Introduction

In ecology, we often measure the effects of a driver on a response to better understand ecological structure and function. There has been increasing recognition that relationships between ecological drivers and their responses are commonly nonlinear^1–9^. Nonlinearity can be quantified by the location of breakpoints (thresholds), where there is an unexpected change in an ecosystem property or process (response) with even a small change in an environmental driver^10–13^. Although both natural and anthropogenic drivers have been linked to threshold responses across a variety of organisms, including macroinvertebrates^4,14^, diatoms^14,15^, fish^16^, and terrestrial plant communities^5^, nonlinearity is a typical response to pressures caused by human activities^17,18^. Nonlinearity is often discovered when breakpoints are accidently crossed. The shift of an aquatic system from an oligotrophic or mesotrophic to eutrophic state, manifested in the sudden rapid increase in algal biomass is one common example which is frequently seen as a result of anthropogenic nutrient contributions^19^, as is the vanishing of coral reefs in favour of brown seaweed caused by declining water quality and overfishing^20^. Disease outbreaks, climate change, and invasive species introductions can cause similar breakpoints in ecosystems and their ecological responses^6,21^. These examples illustrate what are known as regime shifts, where the ecological stressor-response relationship changes at a given breakpoint, resulting in a fundamentally different ecosystem state. Once a system has surpassed its breakpoint and is in a new ecosystem state, returning to the previous state can be extremely difficult^22^, however understanding the nonlinear behaviour of ecological variables can help to find early warning signals of impending regime shifts (catastrophic changes)^23^.

Stream ecosystems are frequently managed from a log-linear framework, thus nonlinearity complicates monitoring, management, and rehabilitation planning^12^. As such, there is a pressing need to better understand how human activities propel ecosystems past breakpoints that lead to ecological regime shifts. Prior to assessing this need, it is important to first quantify breakpoints in the relationships between common variables, such as dissolved nutrients, carbon, other water quality parameters, and ecological responses. There are currently very few ecological breakpoint values published in the literature, although they are beginning to be increasingly reported^17^, likely because our understanding of these relationships has improved and because there are now a variety of statistical tools that allow us to detect ecological breakpoints^10,11,17,24^. The most commonly reported breakpoints are related to nutrients, particularly phosphorus (P), which is often assumed to be the most important nutrient for aquatic ecosystem management. Published total phosphorus (TP) concentration breakpoints for ecological responses (e.g., benthos and algal diversity) of temperate stream and lake ecosystems currently vary several fold^14,17,25,26^. This also appears to be true of total nitrogen (TN)^14,27,28^. There are fewer reported breakpoints for dissolved organic carbon (DOC) in the literature, but the few that are reported are also variable^29–31^. From these few published nutrient and carbon values, there is no one single breakpoint, probably because the critical solute concentrations where ecosystem shifts occur depend on a number of factors, such as geological, ecological, and environmental gradients, including land use.

Anthropogenic land uses, such as urban and agriculture, have long been implicated in ecosystem degradation such as eutrophication through the contribution of nutrients^32,33^. Some evidence has demonstrated that ecological responses shift once anthropogenic disturbance reaches 30–50% agriculture or about 10% urban land coverage^16,18^. While the relationship between land use and nutrient and carbon concentrations is well studied^15,29,34–36^, understanding the influence that land use has on the breakpoints of these solutes may yield useful information for watershed management^12^. If employed as a preventative tool, knowing the land use conditions where ecological breakpoints are likely to occur could compliment regular nutrient and carbon monitoring, allowing watershed managers to take appropriate action before a system undergoes a regime shift. This could significantly lessen the potential ecological and economic burdens associated with abrupt regime shifts and new ecological stable states. Several studies have used breakpoint detection methods to suggest nutrient guidelines to protect ecosystems^14,26,37^, thus continued investigation and application is imperative to determine the feasibility of this emerging approach.

Here, we used previously collected data from three published studies with sites located in southern Ontario, Canada^15,35,36^ to assess the prevalence of nonlinearity and the location of ecological breakpoints. We focused on relationships between three solutes: DOC, total dissolved nitrogen (TDN), and total dissolved phosphorus (TDP) and ecological responses of various stream communities and water quality parameters. Using these data, we had two primary objectives which differed from the original studies; 1) to quantify dissolved solute (nutrient and carbon) breakpoints across a variety of stream community and water quality responses using multiple breakpoint detection methods, and 2) to determine the level of anthropogenic land use associated with above- and below-breakpoint solute concentrations. We expected to identify breakpoints in a consistent range for each solute across methods. We also expected that the percentages of anthropogenic land use would differ depending on whether measured solute concentrations were above- or below-breakpoint, which would suggest a land use effect on ecological regime shifts.

## Methods

Data for the breakpoint analyses were obtained from previously published studies examining the nature of ecosystem, community, and water quality responses to agricultural and urban land use. These studies focussed on both structural and functional responses of bacterial^35^, diatom^15^, and mussel^36^ communities (hereafter referred to as the bacterial, diatom, and mussel datasets) in a network of individual streams located across Ontario, Canada, with datasets consisting of a variety of community and water quality response parameters (Supplemental Tables S1–S3). The number of streams studied varied between datasets: 44 (diatom), 14 (mussel), and 53 (bacterial), but all sites were located within the same sampling network^15,35,36^. The bacterial dataset sites were sampled in late summer/early autumn. The diatom and mussel dataset sites were sampled during summer baseflow and spring/snowmelt conditions, respectively. All data represent single sampling events at each site. In each dataset, stream sites were characterized by a wide diversity of catchment land use and land cover which were primarily dominated by agriculture (up to 87%), wetlands (up to 68%), and/or forests (up to 55%).

We assessed bivariate relationships of water quality and community response parameters with three solutes: TDN, TDP, and DOC. In total, this consisted of 27 relationships each in the bacterial and diatom datasets, and 40 in the mussel dataset. While many of the response variables were similar across the community datasets, such as dissolved organic matter (DOM) composition and physical properties including total suspended solids and conductivity, many parameters were specific to each dataset (Supplemental Tables S1–S3). The difference in parameters reflects, to some degree, the different structural and functional responses of the bacterial, diatom, and mussel communities to stressors. The data of the community and water quality responses were previously published and detailed sample collection methodology, site descriptions, and response variables can be found in the original manuscripts^15,35,36^.

There are multiple methods for estimating breakpoints in bivariate relationships which are subject to their own biases and challenges. We therefore used three methods for a robust estimate of TDN, TDP, and DOC concentration breakpoints. We used piecewise breakpoint regression^17^, two-dimensional Kolmogorov-Smirnov tests (2DKS)^24^, and significant zero crossings (SiZer)^10,11^ to quantify breakpoints. The goal of using multiple techniques was not to critique the methodology (this has been discussed elsewhere)^17^ but to obtain a range of breakpoints as the calculation of breakpoints is sensitive to the test selection and dataset quality^38^. Piecewise regressions were additionally used to assess the prevalence of nonlinearity in our datasets, as this is the only method we employed that could compare linear and nonlinear models.

Piecewise regressions were performed using SegReg software^39^, which uses splines to complete segmented linear regressions, selecting the best breakpoint based on R^2^ values for statistically significant (α = 0.05) relationships. As this analysis is based on linear segments, we normalized the data (log or square root transformations) when necessary to meet the assumptions of a linear regression model. While the other breakpoint analyses we used were non-parametric, we used the transformed data in these for consistency. The piecewise method can detect linear model relationships and five types of nonlinear relationships (two connected sloping line segments, a horizontal segment followed by a sloping segment, a sloping segment followed by a horizontal segment, two disconnected horizontal segments, two disconnected segments where at least one is sloping). Other relationships that did not fit into the linear model or one of the five prescribed nonlinear models were labelled as “undefined.”

2DKS breakpoints were determined using big2dks software^40^. 2DKS identifies breakpoints by assessing significant differences (α = 0.05) in the distributions of the data on either side of potential breakpoints to assess whether they likely came from the same population. The breakpoint is the point at which the distributions are at their maximum cumulative difference, however this method will likely do poorly if there are additional points with the same maximum distance value^41^. SiZer was run using the R^42^ package of that name^43^. Unlike piecewise regression and 2DKS analyses, determining breakpoint values in SiZer is more subjective, and does not give an indication of statistical significance or uncertainty, rather the SiZer output consists of a graphical representation of breakpoint locations across a range of data resolutions (*h* values). It has been recommended that multiple resolutions be explored in order to select the most representative breakpoint^11^ because selecting an *h* value that is too great can result in missed breakpoints due to over-smoothing, while small *h* values may result in under-smoothing, with an over-detection of thresholds. Additionally, SiZer is not always able to estimate a breakpoint value even though it identifies that a breakpoint is present because a relationship can begin to (or continue to) change before (or after) the actual breakpoint is reached, making quantification difficult. Moreover, SiZer is able to identify multiple breakpoints (while piecewise and 2DKS are limited to one). For these reasons, we generally examined three *h* values (low, medium, and high) for each bivariate relationship. We retained the breakpoint value from the resolution that most closely coincided with the breakpoints identified by piecewise and/or 2DKS, which was typically the first breakpoint observed in the SiZer output and was most often associated with a medium resolution. “Secondary” breakpoints (that tended to be different than those detected by the other methods) were also noted. We compared the breakpoints for each solute using two-way ANOVA (and Tukey post-hoc tests) for the breakpoint detection method and dataset factors at an alpha level of 0.05.

To assess the influence that land use had on solute breakpoints, we first reduced the land use variables using principal components analysis (PCA; Supplemental Fig. S1). Land cover was quantified as published in the original studies^15,35,36^. The 1^st^ and 2^nd^ principal component axes (PC1 and PC2) explained 37% and 26% of the variance in the data, respectively. PC1 represented a gradient of forest land cover to agricultural land use, with a strong correlation between PC1 and percent agriculture (linear regression R^2^ = 0.68, p < 0.001, n = 103), while PC2 represented a gradient of wetland to urban land, with a strong percent urban correlation (linear regression R^2^ = 0.85, p < 0.001, n = 103). We then tested whether land use PC axes (scores) that corresponded to the values above and below the previously identified breakpoints differed significantly using Wilcoxon tests at an alpha level of 0.05. This was done only for datasets where all three methods (piecewise, 2DKS, and SiZer) detected a breakpoint. We identified the PC land use value associated with the point at which the probability distributions intersected using the R^42^ package “overlapping^44^”. This can also be thought of as the point at which the above- and below-breakpoint land use distribution differences were maximized. Using the relationship between land use and the PC axes, we calculated the percentage of agricultural or urban land from the PC breakpoint values. This process was completed three times for each solute (once for each dataset), and the land use breakpoints we report are means of these values. We did not do this separately for each breakpoint detection method, as ANOVA with Tukey post-hoc tests could not, for the most part, detect significant differences between the methods used (described in the Results section; Table 1).

**Table 1:** ANOVA test statistics and significance levels for the differences between mean breakpoints for each solute.

Solute	N	Dataset	Method	Dataset × Method (Interaction)
DOC	*122*	37.5***	3.5*	4.0**
TDP	*123*	199.3***	*0.9*	3.1*
TDN	*139*	6.7**	5.2**	*2.1*

Datasets include the bacterial, diatom, and mussel parent datasets, and methods include piecewise regression, 2DKS, and SiZer. Significance levels are as follows: *p < 0.05, **p < 0.01, ***p < 0.001.

## Results

**Figure 1:**
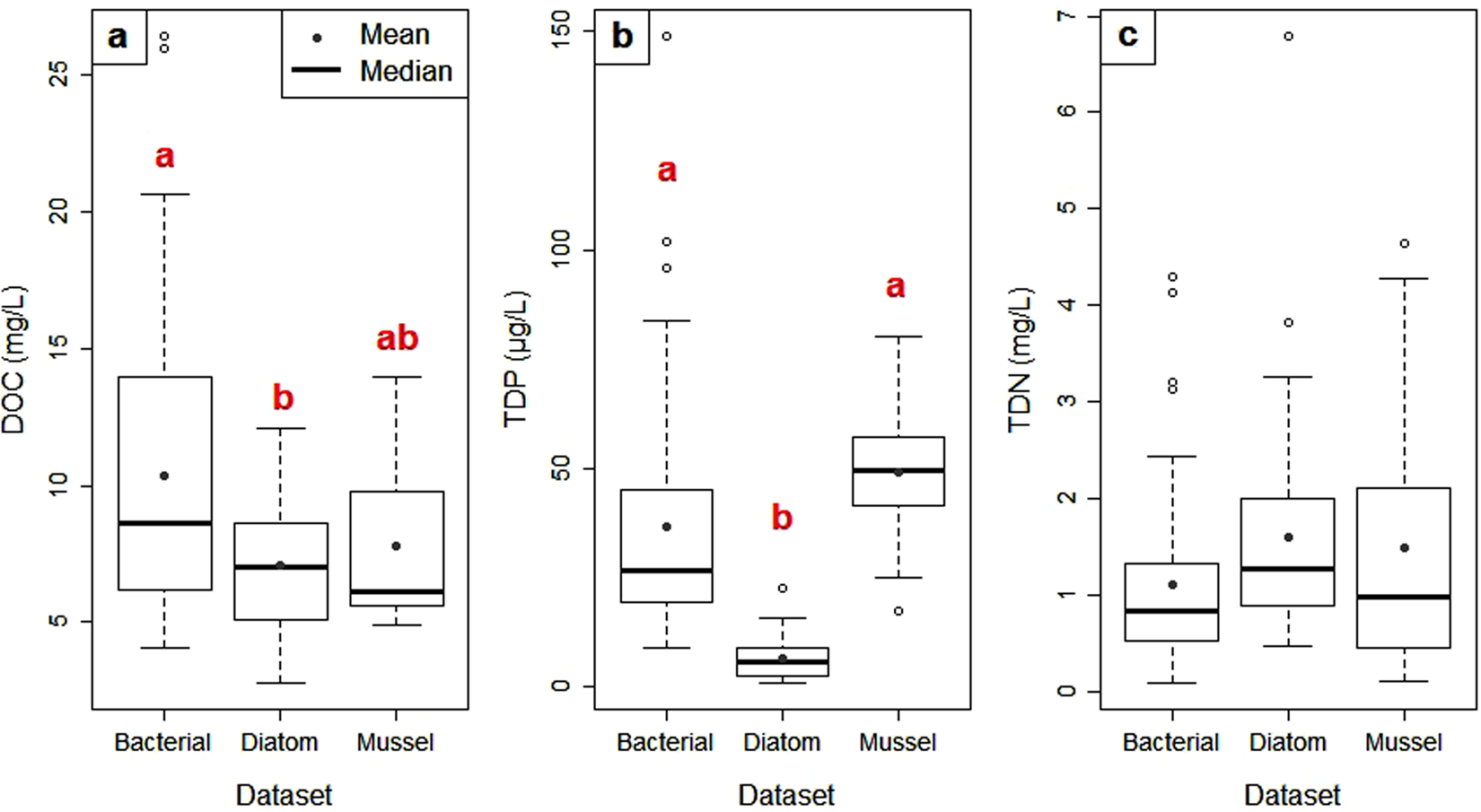
Measured (**a**) DOC, (**b**) TDP, and (**c**) TDN ranges for the community and water quality responses from the bacterial, diatom, and mussel datasets. Whiskers represent 1.5 times the interquartile range. Solid lines represent medians, while grey dots denote means. Red letters indicate significant difference between datasets (ANOVA p < 0.05).

Breakpoints were prevalent in the bivariate relationships between dissolved solute concentrations and ecological responses, but varied depending on the dataset examined, possibly due to differences in measured concentration ranges. Measured DOC concentration ranged from 4 to 14 mg C/L, depending on the dataset, however the diatom dataset had significantly lower concentrations compared to the bacterial dataset (Fig. 1a). The diatom dataset also had significantly lower TDP concentrations than the other datasets (Fig. 1b), while TDN concentrations were similar across all datasets (Fig. 1c). TDP and TDN ranged from 0.9 to 149 μg P/L and 0.08 to 6.8 mg N/L, respectively.

**Figure 2:**
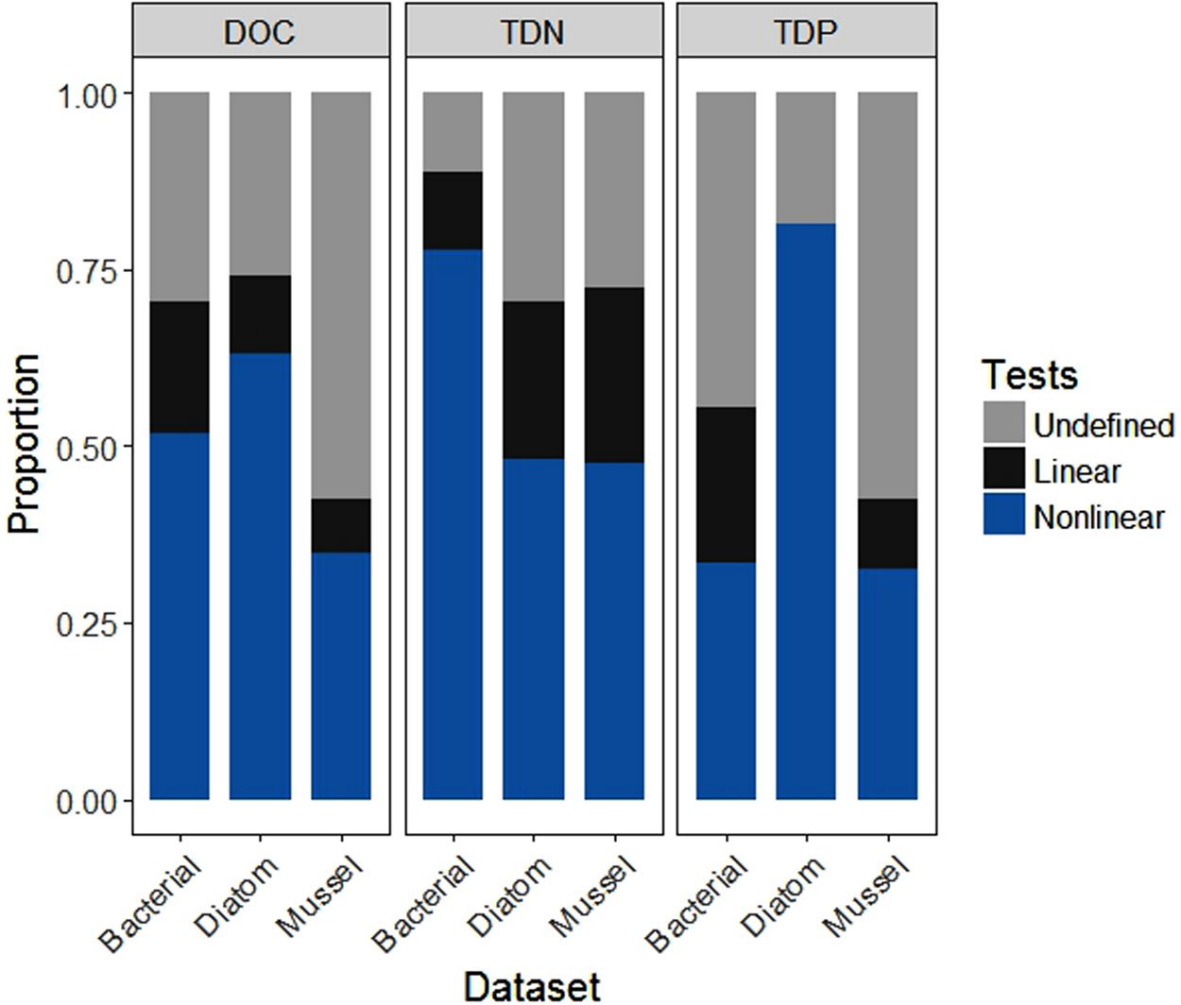
The proportion of community and water quality responses to each solute in the bacterial, diatom, and mussel datasets that piecewise regressions identified as nonlinear, linear, and undefined.

**Figure 3:**
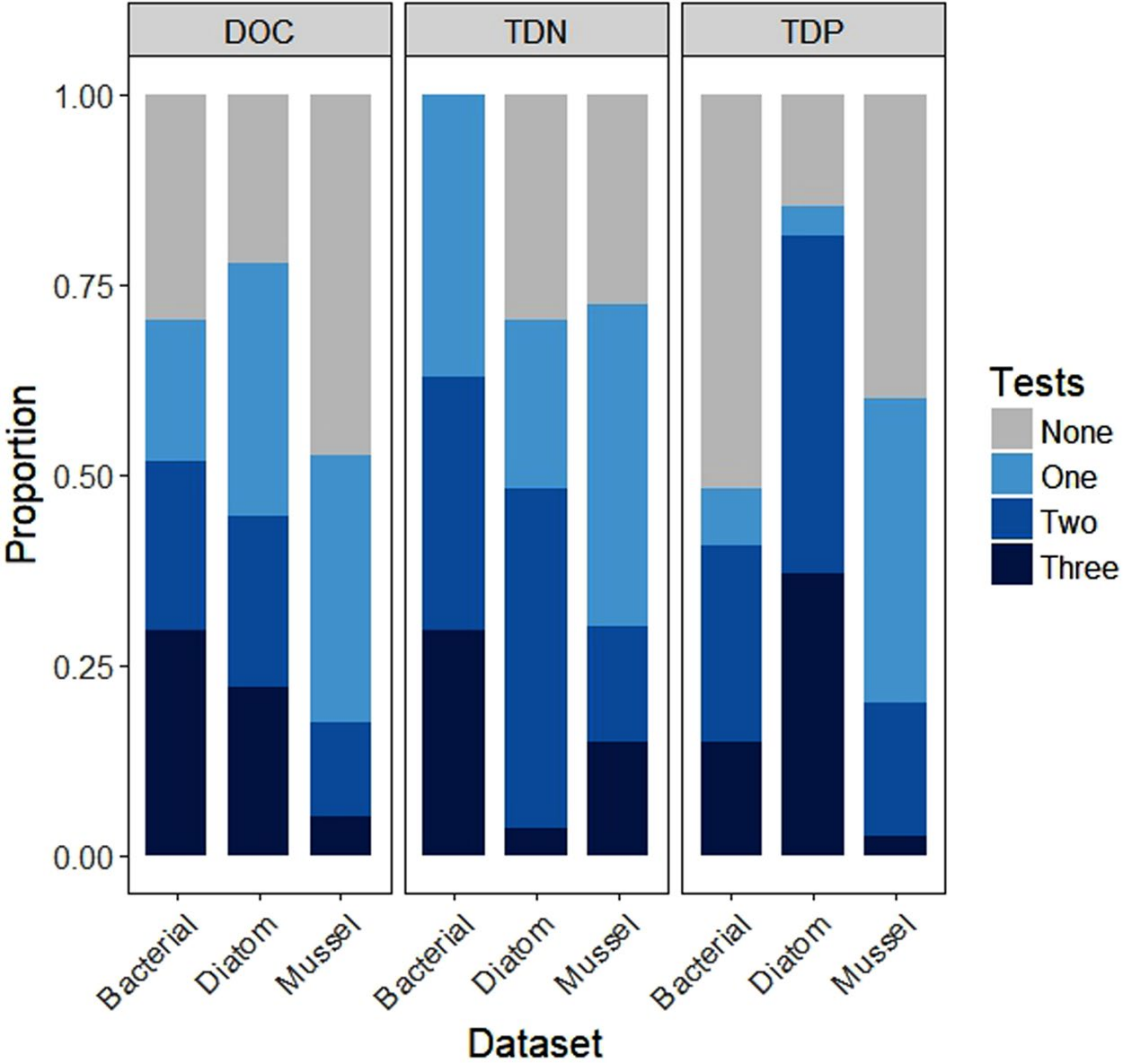
The proportion of community and water quality responses to each solute in the bacterial, diatom, and mussel datasets in which one, two, three, or no breakpoint detection method identified a breakpoint.

**Figure 4:**
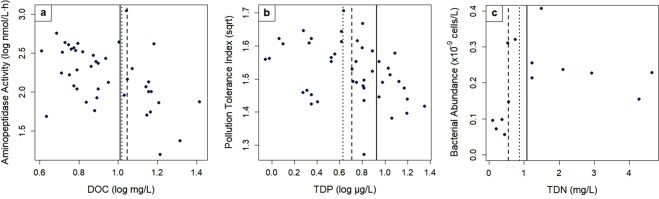
Examples of bivariate relationships with breakpoints determined by piecewise regression (short dashed lines), 2DKS (long dashed lines), and SiZer (solid lines) using data from the (**a**) bacterial, (**b**) diatom, and (**c**) mussel datasets. Breakpoint values for all bivariate relationships are provided in Supplemental Tables S1–S3.

**Figure 5:**
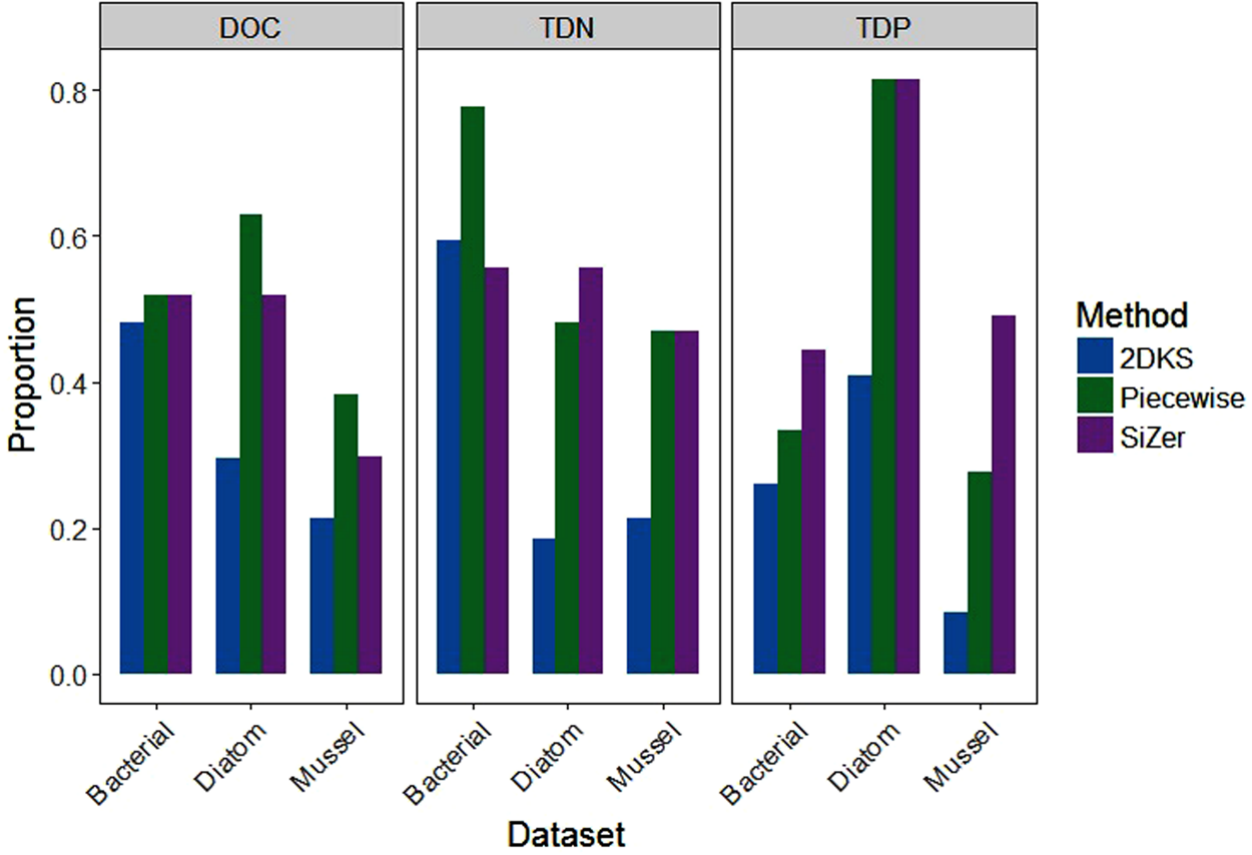
The proportion of bivariate solute-community and water quality parameter relationships where a breakpoint was identified by each method for each parent dataset (bacterial, diatom, and mussel) and solute.

Overall, we tested 94 bivariate relationships per solute and using piecewise regression we found that nonlinearity was present in 52% of all relationships, whereas 14% of datasets tested were linear, on average (Fig. 2). 34% of the relationships we tested were categorized as “undefined,” which means that they did not fit one of the piecewise method’s five prescribed nonlinear models, nor the linear model. At least one of the breakpoint detection methods identified a breakpoint in about half or more of the bivariate relationships, but relationships where all three methods were in agreement about the presence of a breakpoint were rarer at only 2.5% to 37%, depending on the dataset and solute (Fig. 3). Examples of bivariate relationships with breakpoint locations are provided in Fig. 4 with all breakpoint values available in Supplemental Tables S1–S3. There was no apparent pattern in the number of methods that identified breakpoints, although we did find that breakpoint detection across all methods tended to be lowest for the mussel dataset (Fig. 5). We noted that 2DKS was generally not able to identify breakpoints as frequently as piecewise regressions or SiZer, and that SiZer consistently detected more breakpoints for TDP bivariate relationships (except for piecewise regression in the diatom dataset, which detected a similar proportion). Similarly, for DOC, piecewise regression found more breakpoints, but no consistent pattern was found for TDN.

**Figure 6:**
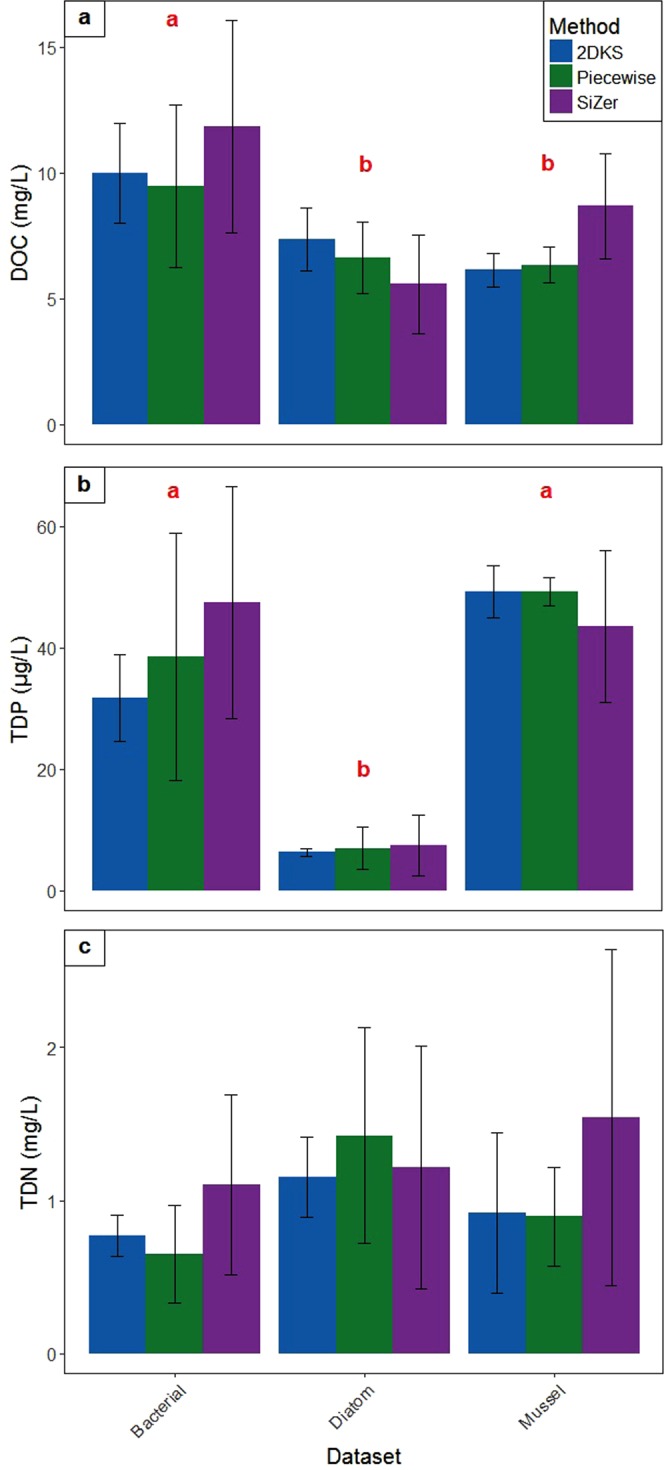
Mean breakpoints in the relationships between (**a**) DOC, (**b**) TDP, and (**c**) TDN and community and water quality responses for each breakpoint detection method in each datasets (bacterial, diatom, and mussel). Error bars represent standard deviation. Red letters denote general significant differences between datasets (ANOVA p < 0.05).

Within each dataset, mean breakpoint values were similar across all detection methods within each solute in all but a single case. Statistical differences between mean breakpoints within each dataset only occurred between SiZer and 2DKS in the TDP bacterial dataset (Table 1, Fig. 6), but as this was the only instance of this effect, and given that neither of these methods’ mean breakpoint significantly differed from that identified by piecewise regression, we combined mean TDP breakpoints for this dataset as we did for all other solutes and datasets.

Regarding DOC, mean breakpoints were significantly higher in the bacterial dataset, at approximately 10.4 mg C/L, compared to both the diatom and mussel datasets, at about 6.7 mg C/L (Fig. 6a). When considered against our entire range of measured DOC concentrations (across all datasets, Fig. 1a), the lower breakpoint occurred in the first 17% of this range, while the upper breakpoint occurred at about 32%. In the bacterial dataset, we noted that SiZer, which is the only method that was able to detect multiple breakpoints, also detected lower breakpoints (between 4.7 and 7.4 mg C/L) in about 18% of the datasets. SiZer also identified low “secondary” breakpoints ( < 5 mg C/L) in 40% of bivariate relationships in the diatom dataset. All breakpoints ranged from about 3–22 mg C/L, which overlapped 79% of the measured DOC range. TDP breakpoints were significantly lower for the diatom dataset (Fig. 6b), with a mean breakpoint concentration of about 6.9 μg P/L (at 4% of the measured range of TDP across all datasets). The bacterial and mussel datasets had an average breakpoint of about 43 μg P/L (at 28% of the TDP range). We calculated individual breakpoints of 1–85 μg P/L, overlapping 57% of the measured TDP concentrations. Mean breakpoints for TDN were similar across all datasets, with an average of 1.07 mg N/L, or at about 13% of our measured TDN range (Fig. 6c). TDN breakpoints were between about 0.2 and 3.8 mg N/L, which overlapped with the measured TDN range by 54%.

**Figure 7:**
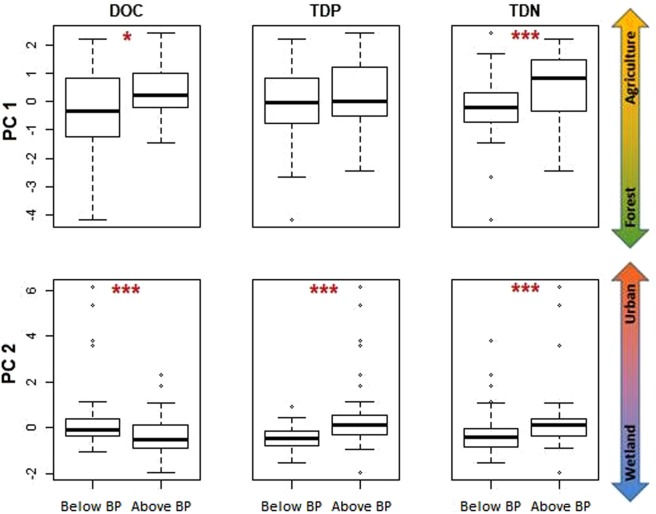
Land use gradient (from principle components analysis) distributions for below- and above-breakpoint (Below BP and Above BP, respectively) concentrations for each solute using bivariate relationships in which all three breakpoint detection methods agreed on the presence of a breakpoint. Whiskers represent 1.5 times the interquartile range, while solid bars represent median values. Significance levels are as follows: *p < 0.05, **p < 0.01, ***p < 0.001 (Wilcoxon tests).

By grouping DOC, TDP, and TDN concentrations by whether they occurred below or above their dataset-specific mean breakpoints, we detected significant differences in the land use associated with the sites where the samples were taken. We found that TDN and DOC breakpoints were significantly associated with higher levels of agriculture than below-breakpoint concentrations (Wilcoxon test, p < 0.05, n = 108; Fig. 7). We calculated that the percentage of agricultural land use associated with DOC and TDN breakpoints was 34.0 ± 0.4% and 42.7 ± 1.3%, respectively. PC1 land use was not significantly different between above- and below-breakpoint TDP measurements (Wilcoxon test, p > 0.05, n = 108). For PC2 land use (wetland-urban gradient), we found a significant difference between below- and above-breakpoint nutrients with more urban land use at sites with above-breakpoint measurements, while the opposite was true for DOC. We calculated the level of urban land use associated with TDP and TDN breakpoints to be 9.4 ± 0.5% and 9.5 ± 0.4%, respectively, while DOC breakpoints occurred at 15.4 ± 0.2% wetland. When at least one of our breakpoint methods detected a breakpoint (a more inclusive approach) agricultural breakpoints for DOC and TDN occurred at 34.0 ± 0.5% and 43.2 ± 1.1% land use, respectively. Urban breakpoints for TDP and TDN occurred at 9.5 ± 0.5% and 9.6 ± 0.2% land use, respectively, and a wetland breakpoint for DOC was found at 15.4 ± 0.3% catchment land cover (Supplemental Fig. S2).

## Discussion

We found that nonlinearity was prevalent, being detected, on average, in more than half of all tested solute-ecological responses by piecewise regression. Using data from all three breakpoint detection methods, DOC and TDP relationships with ecological responses were associated with two breakpoints, whereas only one breakpoint was identified for responses tested against TDN, which highlights the potential complexity of nonlinear relationships across large ranges of solutes. We found that the forest land cover to agricultural land use gradient was associated with a TDN and DOC breakpoint of about 34–43% catchment agriculture, whereas a relatively small amount of urban land use and wetland cover was associated with nutrient and DOC breakpoints, respectively. Our study not only quantifies stream solute breakpoints across a variety of ecological and water quality responses, but also highlights the contribution of anthropogenic activities to ecological breakpoints and potential regime shifts.

We found that, on average, 52% of all stream water quality and community structural and functional responses to DOC, TDP, and TDN concentrations were nonlinear (Fig. 2). This percentage may be higher given that the piecewise regression model only fit the data to five possible nonlinear models, thus it is probable that at least some of the “undefined” relationships would fit other nonlinear models. Regardless, this high prevalence of nonlinearity is consistent with previous work suggesting nonlinearity is common in nature^6,9,17,19^.

There was evidence of multiple DOC breakpoints in relationships with stream water quality and community responses. In the literature, DOC breakpoints are rarely reported, relatively variable and, to our knowledge, multiple breakpoints have not been reported for DOC or for nutrients. D’Amario & Xenopoulos^29^, who studied CO_2_ in this same stream network, reported a DOC breakpoint of about 7 mg C/L, which is relatively close to our lower reported breakpoint value (6.7 mg C/L; Fig. 6a). Other studies report breakpoints in lakes of about 4 mg C/L for chironomid composition^31^, and 4.8 mg C/L for light attenuation^30^. We did not find this lower breakpoint value (though there was some evidence of it) potentially due to the scarcity of data at the lower range^30^, typical in streams. Greater breakpoints, similar to the 10.4 mg C/L we report here, have been associated with the effects of DOC on metal toxicity to organisms, with breakpoints reported around 8.7 mg C/L^45^ and 11 mg C/L^46^. We cannot comment on whether metal toxicity contributed to the shift in our DOC relationships as metal concentrations were not measured in any of our parent studies. The presence of two breakpoints in the data suggests that nonlinearity within ecological data can be complex, consisting of multiple thresholds, but it may also be the result of temporal variation between datasets. Our analysis did not capture important temporal variation in stream flow, turbidity, and water temperature, all of which can influence DOC concentration. Nevertheless, SiZer detected a lower breakpoint even when the DOC concentration range was greatest (in the late summer/early autumn sampling; Fig. 1a), lending some support to the argument that multiple, somewhat consistent breakpoints are likely to exist in river systems.

We found that DOC breakpoints and concentrations were related to land use gradients. DOC concentrations were below-breakpoint up to about 34% catchment agriculture (Fig. 7), which is in line with land use breakpoints reported by other studies for various community and hydrological parameters^18^. DOC was additionally above-breakpoint up to about 15% wetland coverage (Fig. 7). Both wetland and agricultural streams typically have greater DOC concentrations than urban and forested streams, respectively^29,34^, thus these land use percentages may be the critical values at which concentrations exceed their breakpoints in these catchments (Fig. 6a). Indeed, increasing DOC concentrations, which at first promote primary productivity by providing a supply of carbon, could eventually cause light limitation through increased turbidity^30^, potentially causing a shift in algal communities and may partially explain the land use (agriculture and wetland) effect. However, the differences in DOC concentration related to land use are minor relative to the differences in DOM composition (quality), which were among the response variables used in our study. The DOM pool is composed of more labile and less complex carbon moieties within agricultural and urban streams compared to forested and wetland streams, which generally contains more complex, humic, and refractory carbon^29,35,47,48^. These differences in DOM composition are likely responsible for changes in microbial community structure and function^35,49^ resulting in abrupt changes to decomposition, respiration, and the food web in general. Taken together, it may be that these microbial and algal community changes, along with other land use related factors (canopy cover, ions, flow regime, etc.) reach a critical point when DOC concentrations reach about 6.7 mg C/L and 10.4 mg C/L. At these breakpoints, a regime shift may occur due to a positive feedback loop. For example, Williams *et al*.^35^ suggested that degraded riparian buffers in anthropogenic catchments would increase microbial DOM processing, creating even more labile DOM which would further increase processing rates. Positive feedback loops such as this would create a new ecosystem stable state, preventing it from returning to its previous state.

We identified a single breakpoint for TDN at approximately 1 mg N/L (Fig. 6c). We were not able to find TDN breakpoints reported in the literature, however total nitrogen (TN) breakpoints were in the 0.47 to 1.79 mg N/L range^14,27,28^. The measured TN concentration ranges of these studies were similar to, or exceeded, our TDN concentration range, yet no high-concentration TN breakpoint was observed^14,27,28^. This, in conjunction with our data, suggests that, unlike DOC, TDN relationships with stream response parameters have a single breakpoint, which we suspect may be related to the contribution of NO_3_ to TDN. While NO_3_ data was not recorded in the datasets used in our study, it was available from a previous research study in this same stream network^29^. We briefly tested the ratio of NO_3_ to TDN above and below the 1 mg N/L breakpoint with the data from that study and found that the percentage of NO_3_ in TDN was significantly higher when TDN concentrations were above the breakpoint (67%) compared to below (29%; ANOVA, N = 113, F = 3.93, p < 0.0001), though more research would be required to determine if NO_3_ is indeed contributing to the breakpoint in our study.

Similar to DOC, TDN concentrations tended to be above-breakpoint over about 43% agricultural land use (Fig. 7). We know that TDN concentrations increase with agriculture in these catchments^29,36,47^, but this may mean that at the 43% agriculture mark this TDN is composed of excess NO_3_. Different taxa of phytoplankton respond differently to increases in available N, therefore a greater proportion of NO_3_ can result in changes to the composition of the algal community^50^. A shift in the proportion of available N in the stream may also cause aquatic plants and algae nutrient ratios to change, which can potentially influence grazing behaviour in aquatic invertebrates^51^ with cascading effects through the food web.

We found two TDP breakpoints at about 6.9 µg P/L and 39–47 µg P/L (depending on the dataset; Fig. 6b). Phosphorus breakpoints have been reported in the literature for freshwater ecosystems, with values for total phosphorus (TP) ranging from 5–50 µg P/L for ecological community responses^14,26,28,37,52,53^. As with TDN, we were not able to find TDP breakpoint values in the literature, however our average breakpoints did not exceed reported TP breakpoints, and are therefore likely reasonable. The large range in the TP breakpoint values in the literature may be partially due to different TP concentration ranges, but also may be due to differences in the fraction of TDP. Here, we report on TDP, as it is more immediately bioavailable, with greater impacts on eutrophication than TP. TDP breakpoints are perhaps the most important of those we examined in this study, as P is managed as the limiting nutrient in many temperate freshwater systems, with elevated concentrations associated with algal blooms^33,54^. Dodds *et al*.^55^ suggested that TP above 25 µg P/L pushes a stream towards mesotrophic conditions, and above 75 µg P/L to eutrophic. As discussed by Folke *et al*.^22^, elevated lake concentrations of P are accompanied by abundant algae and high sediment P recycling; a positive feedback system that is difficult to return from. It is possible that our TDP breakpoints may indicate similar regime shifts, however further research into the trophic status associated with below- and above-breakpoint conditions is required to determine if this is true in our study streams.

The occurrence of multiple TDP breakpoints in our study may be related to the large TDP ranges we tested (Fig. 1b) and/or may be due to the temporal variation that drives TDP in streams. For example, TDP is highly mobile during flooding^56,57^, thus during spring melt stream concentrations can be elevated. This was also observed in our data, where the dataset sampled during high flows in spring (mussel) and during baseflow (diatom) showed the highest and lowest TDP concentrations, respectively (Fig. 1b). These different breakpoints, therefore, may be the result of seasonal flow regimes. We were unable to find evidence of both breakpoints in a single dataset (due to the lack of overlap in the concentration ranges), so while TDP may contain multiple, consistent breakpoints regardless of flow, more research is required to confirm this.

TDN and TDP concentrations at our study sites were also above-breakpoint when urban land made up at least 9.5% of catchment land cover (Fig. 7). Although this is a relatively low proportion of land cover, at this level TDN was above ~1 mg N/L and TDP was above 6.9 µg P/L and 39–47 µg P/L (Fig. 6b,c). We know that urban land use is often associated with increased imperviousness, degraded riparian buffers, and additional nutrient and pollutant inputs, largely from point sources such as wastewater^58–60^. Thus, it is not surprising that nutrient concentrations were greater at sites with even a small amount of urban land coverage (Fig. 7). Other studies also report that a low proportion of urban land use, particularly impervious surface area, can have relatively large impacts on stream communities^16,18,61^. As with agriculture, the cause for the breakpoints we observed would be complex, and likely involve ecological-community shifts. Chloride, for example, which can be contributed through road salts, has been shown to shift the diatom community species composition in these streams^15^, and other pollutants likely shift macroinvertebrate assemblages as well, which would cause changes to the food web. Nutrient availability, particularly P, can also shift algal community species composition^15^, and plant nutrient and carbon ratios can shift based on available material, as previously discussed.

It is important to note that while we report breakpoints for DOC, TDN, and TDP for these Ontario catchments and relate these to land use, our results are likely site-specific. It is highly probable that other environments will have different breakpoints, as dictated by the catchment’s land cover, overall climate, and underlying geology. For example, desert streams can be N limited^62^, thus there may be a threshold of land cover in these types of watersheds that are related to an N breakpoint different than the one we report here. Since stream communities will likely vary depending on the geographic location of the catchment, we expect that nutrient and carbon breakpoints will differ across environments.

While the goal of our study was not to critique the various breakpoint detection methods used, it is noteworthy that the outcome of our conservative (all three methods found a breakpoint) and inclusive (one or more methods found a breakpoint) land use analyses were nearly identical (Fig. 7, Supplemental Fig. S2), with less variation in the more conservative method. This is probably because the average breakpoints were similar across methods. We suggest, therefore, that although using multiple methods would be ideal, employing even a single breakpoint detection method in this type of analysis can still be useful for estimating solute breakpoints and land use thresholds.

Quantifying land use thresholds associated with ecological breakpoints can be useful for management agencies assessing ecosystem health to maintain biodiversity and ecological function. Knowing the proportion of land use where shifts in ecological relationships occur, along with the use of common solute indicators such as dissolved nutrients and carbon, may be essential for recognizing warning signals for better watershed management planning. Here, we found evidence of multiple breakpoints for both DOC (6.7 and 10.4 mg C/L) and TDP (6.9 and 43 μg P/L) in solute-ecological response relationships, and a single breakpoint for TDN around 1 mg N/L. We report that about 34–43% agricultural land use or 9.5% urban land use in a catchment is sufficient to change the nature of ecological responses to solutes. Wetland cover was particularly important for DOC, where the nature of the relationship between DOC and ecological responses and water quality seem to shift at a breakpoint of 15%. Overall, this information can be helpful for assisting watershed managers in setting criteria and guiding restoration and monitoring efforts. This knowledge can be applied to other systems, taking site-specific factors into account, in order to identify streams that may be at risk of an ecological regime shift.

## Supplementary information

Supplemental Information
