# Supplementary material for: The prevalence of nonlinearity and detection of ecological breakpoints across a land use gradient in streams
Source: Sci Rep. 2019 Mar 7;9:3878. doi: 10.1038/s41598-019-40349-4 (PMC6406005; doi:10.1038/s41598-019-40349-4)
Supplement: Supplementary file 1 — Supplemental Information [file 41598_2019_40349_MOESM1_ESM.pdf]

The prevalence of nonlinearity and detection of ecological breakpoints across a land use gradient in streams

Sarah C. D'Amario[1], Daniel C. Rearick[2], Christina Fasching[1], Steven W. Kembel[3], Emily Porter-Goff[1], Daniel E. Spooner[4], Clayton J. Williams[5], Henry F. Wilson[6] and Marguerite A. Xenopoulos[1]

[1]Department of Biology, Trent University, Peterborough, ON, Canada

[2]Environmental and Life Sciences Graduate Program, Trent University, Peterborough, ON, Canada

[3]Département des sciences biologiques, Université du Québec à Montréal, Montréal, QC, Canada

[4]Department of Biology, Lock Haven University

[5]Rubenstein School of Environment and Natural Resources, University of Vermont, Burlington, VT

[6]Agriculture and Agri-Food Canada, Brandon, MB, Canada

Supplemental Table S1. DOC breakpoint values (mg/L) for all parameters used in analysis. Breakpoint methods include SiZer (SZ), 2DKS, and piecewise (PW) regression. Dashes indicate that no breakpoint was detected, while empty cells indicate that no data was present for that parameter.

Type	Parameter	Unit	Description	Bacterial Dataset			Diatom Dataset			Mussel Dataset		
				SZ	2DKS	PW	SZ	2DKS	PW	SZ	2DKS	PW
Dissolved organic matter optical properties	Component 1	%	Parallel factor analysis (PARAFAC) component 1*	9.65	7.70	10.09				6.45	5.95	-
	Component 2	%	PARAFAC component 2*	21.94	-	-				-	-	5.68
	Component 3	%	PARAFAC component 3*	-	-	-				-	-	-
	Component 4	%	PARAFAC component 4*	10.21	9.78	11.21				-	-	-
	Component 5	%	PARAFAC component 5*	9.65	7.90	-				10.09	5.95	7.68
	Component 6	%	PARAFAC component 6*	-	-	-				-	-	-
	Component 7	%	PARAFAC component 7*	11.32	-	-				-	-	-
	Component 8	%	PARAFAC component 8*	7.41	10.73	14.34				10.09	6.21	7.32
	Component 9	%	PARAFAC component 9*	17.47	11.15	14.12				10.09	7.91	7.32
	β:α	Ratio	Ratio of 2 known fluorescence values*	7.12	10.73	7.59	4.46	8.40	-	5.55	-	6.92
	E_{280}	L/ mg·cm	Coefficient of 280nm molar absorbance*	13.58	10.73	-				-	-	5.77
	Fluorescence index	Index	Fluorescence index*	-	-	5.40	5.99	-	5.75	-	5.64	5.68
	Humification index	Index	Humification index*	8.19	11.15	7.41	3.20	-	-	10.32	5.95	7.68
	Redox index	Index	Redox index*	-	-	-	3.44	7.25	4.79	-	-	-
	Slope ratio	Ratio	Slope ratio*	-	7.47	7.64	-	-	-	8.73	6.21	5.95
	$SUVA_{254}$	L/ mg·cm	Coefficient of 254nm molar absorbance*	15.78	13.83	-	3.57	8.43	6.46	8.73	-	5.77

**Microbial biomass and activity**	α glucosidase	nmol/ L·hr	Alpha glucosidase activity	-	-	9.94						
	β glucosidase	nmol/ L·hr	Beta glucosidase activity	-	-	-						
	AMA	nmol/ L·hr	Aminopeptidase activity	10.27	11.15	10.47				5.85	-	-
	APA	nmol/ L·hr	Alkaline phosphatase activity	-	-	14.79				-	-	-
	Bacterial Growth	/day	Doubling time	-	-	-				-	-	-
	Bacterial production	fg C/ cell·day	Bacterial production standardized per cell	-	-	-				-	-	-
	Total bacterial abundance	$\times 10^9$ cells/L	Density of bacteria in stream water	9.43	6.86	7.41				-	-	6.23
	FungalB	mg/cm2	Fungal biomass on leaf litter as measured using ergosterol concentration							-	-	5.75
**Nutrient content & ratios**	$\delta^{15}N$	‰	Mussel tissue $^{15}N:^{14}N$							-	-	-
	C:N	Ratio	Mussel tissue C:N							-	-	5.75
	C:P	Ratio	Mussel tissue C:P							-	-	-
	N:P	Ratio	Mussel tissue N:P							-	-	5.75
	O:N	Ratio	Mussel tissue O:N							-	-	-
	N excretion	µg NH_3/ hr·g/dwt	Mussel N excretion							-	-	-
	P excretion	µg NH_3/ hr·g/dwt	Mussel P excretion							-	-	-
	N:P excretion	Ratio	Mussel N:P excretion							-	-	-
	Periphyton C	mg/cm^2	Periphyton C conc.				-	-	6.76			
	Periphyton N	mg/cm^2	Periphyton N conc.				-	-	-			
	Periphyton P	mg/cm^2	Periphyton P conc.				6.95	-	-			
	Periphyton C:N	Ratio	Periphyton C:N ratio				-	-	5.01			
	Periphyton C:P	Ratio	Periphyton C:P ratio				-	-	5.75			
	Periphyton N:P	Ratio	Periphyton N:P ratio				-	8.40	-			

**Respiration & decay rates**	Brick respiration	mg/L·hr	Periphyton respiration on brick substrate							10.44	-	-
	Disk respiration	mg/L·hr	Periphyton respiration on a silicate disk substrate							6.85	-	-
	Wood respiration	mg/L·hr	Respiration of periphyton on wood substrate							12.56	-	-
	Leaf decay	mg/day	Rate of leaf-litter decay in stream water							-	-	-
	Wood decay	mg/day	Rate of wood decay in stream water							-	5.76	-
**Various indices**	Pigment dissimilarity index	Index	Pigment difference between mussel shell and rock substrate							-	-	6.31
	Macroinvertebrate dissimilarity index	Index	Macroinvertebrate difference between mussel shell and rock substrate							-	-	-
	Pigment diversity – mussel shell	Index	Diversity of pigments on mussel shell							6.85	-	6.03
	Pigment diversity – rock substrate	Index	Diversity of pigments on rock substrate							8.92	-	6.03
	Macroinvertebrate diversity – mussel shell	Index	Diversity of macroinvertebrates on mussel shell							-	-	-
	Macroinvertebrate diversity – rock substrate	Index	Diversity of macroinvertebrates on rock substrate							-	-	-
	Pollution tolerance index	Index	Diatom pollution tolerance				-	-	-			
	Specific conductance index	Index	Specific conductance index of stream water				6.70	-	5.89			
	Trophic diatom index	Index	Trophic diatom index				-	-	-			

**Diversity indices**	Evenness	Index	Diatom species evenness				5.57	-	5.50			
	Richness	Index	Diatom species richness				4.14	5.75	5.50			
	Shannon-Weiner diversity index	Index	Shannon-Weiner diversity index of diatoms				4.80	5.49	5.50			
	Simpson diversity index	Index	Simpson diversity index of diatoms				5.17	-	5.25			
**Physical and water quality**	Chlorophyll a	ng/L	Stream water concentration of chlorophyll a				-	-	8.51			
	Conductivity	µS/cm	Conductivity (mussel dataset) and specific conductivity (bacterial and diatom datasets) of stream water	13.55	10.73	-	-	-	8.51	-	5.95	-
	Dissolved oxygen	mg/L	Stream water concentration of dissolved oxygen				5.57	6.76	7.59			
	Salinity	ppt	Stream water salinity concentration				-	-	8.51			
	Total suspended solids	mg/L	Total suspended solids in stream water	-	-	-	8.36	8.40	8.51	-	5.76	-

Supplemental Table S2. TDP breakpoint values (μg/L) for all parameters used in analysis. Breakpoint methods include SiZer (SZ), 2DKS, and piecewise (PW) regression. Dashes indicate that no breakpoint was detected, while empty cells indicate that no data was present for that parameter.

Type	Parameter	Unit	Description	Bacterial Dataset			Diatom Dataset			Mussel Dataset		
				SZ	2DKS	PW	SZ	2DKS	PW	SZ	2DKS	PW
**Dissolved organic matter optical properties**	Component 1	%	Parallel factor analysis (PARAFAC) component 1*	-	-	-				42.45	-	-
	Component 2	%	PARAFAC component 2*	-	-	67.84				31.46	-	-
	Component 3	%	PARAFAC component 3*	-	-	-				70.70	-	-
	Component 4	%	PARAFAC component 4*	40.59	29.07	34.31				34.60	-	-
	Component 5	%	PARAFAC component 5*	-	-	-				-	-	-
	Component 6	%	PARAFAC component 6*	40.59	29.07	38.50				-	-	-
	Component 7	%	PARAFAC component 7*	-	-	-				-	-	-
	Component 8	%	PARAFAC component 8*	-	-	-				37.74	-	-
	Component 9	%	PARAFAC component 9*	-	-	-				58.14	48.01	48.73
	β:α	Ratio	Ratio of 2 known fluorescence values*	-	-	-	8.47	-	10.72	40.23	-	-
	E_{280}	L/ mg·cm	Coefficient of 280nm molar absorbance*	79.50	22.60	13.80				42.45	-	49.35
	Fluorescence index	Index	Fluorescence index*	-	-	-	5.20	-	8.51	39.31	-	-
	Humification index	Index	Humification index*	-	-	-	7.20	-	7.76	33.03	-	-
	Redox index	Index	Redox index*	42.45	38.26	-	-	-	-	-	-	-
	Slope ratio	Ratio	Slope ratio*	44.09	44.07	-	7.81	-	9.12	-	-	-
	$SUVA_{254}$	L/ mg·cm	Coefficient of 254nm molar absorbance*	85.24	-	25.70	1.81	-	2.60	42.45	-	49.35

**Microbial biomass and activity**	α glucosidase	nmol/ L·hr	Alpha glucosidase activity	-	-	-						
	β glucosidase	nmol/ L·hr	Beta glucosidase activity	47.58	-	48.28						
	AMA	nmol/ L·hr	Aminopeptidase activity	-	-	-				-	-	47.86
	APA	nmol/ L·hr	Alkaline phosphatase activity	29.96	29.07	70.79				27.43	-	-
	Bacterial Growth	/day	Doubling time	-	-	-				-	-	-
	Bacterial production	fg C/ cell·day	Bacterial production standardized per cell	-	-	-				-	-	-
	Total bacterial abundance	$\times 10^9$ cells/L	Density of bacteria in stream water	27.94	29.84	-				-	-	-
	FungalB	mg/cm2	Fungal biomass on leaf litter as measured using ergosterol concentration							-	-	48.98
**Nutrient content & ratios**	$\delta^{15}N$	‰	Mussel tissue $^{15}N:^{14}N$							30.77	-	48.98
	C:N	Ratio	Mussel tissue C:N							-	-	-
	C:P	Ratio	Mussel tissue C:P							48.71	45.16	47.86
	N:P	Ratio	Mussel tissue N:P							24.46	-	-
	O:N	Ratio	Mussel tissue O:N							-	-	45.71
	N excretion	μg NH_3/ hr·g/dwt	Mussel N excretion							-	48.73	-
	P excretion	μg NH_3/ hr·g/dwt	Mussel P excretion							-	55.15	50.12
	N:P excretion	Ratio	Mussel N:P excretion							-	-	-
	Periphyton C	mg/cm^2	Periphyton C conc.				-	-	-			
	Periphyton N	mg/cm^2	Periphyton N conc.				2.13	6.50	2.29			
	Periphyton P	mg/cm^2	Periphyton P conc.				-	-	-			
	Periphyton C:N	Ratio	Periphyton C:N ratio				5.20	6.40	3.09			
	Periphyton C:P	Ratio	Periphyton C:P ratio				-	-	-			
	Periphyton N:P	Ratio	Periphyton N:P ratio				4.80	7.70	3.09			

**Respiration & decay rates**	Brick respiration	mg/L·hr	Periphyton respiration on brick substrate							-	-	-
	Disk respiration	mg/L·hr	Periphyton respiration on a silicate disk substrate							56.77	-	-
	Wood respiration	mg/L·hr	Respiration of periphyton on wood substrate							-	-	47.86
	Leaf decay	mg/day	Rate of leaf-litter decay in stream water							68.75	-	52.48
	Wood decay	mg/day	Rate of wood decay in stream water							29.62	-	-
**Various indices**	Pigment dissimilarity index	Index	Pigment difference between mussel shell and rock substrate							43.43	-	-
	Macroinvertebrate dissimilarity index	Index	Macroinvertebrate difference between mussel shell and rock substrate							-	-	-
	Pigment diversity – mussel shell	Index	Diversity of pigments on mussel shell							-	-	-
	Pigment diversity – rock substrate	Index	Diversity of pigments on rock substrate							46.88	-	-
	Macroinvertebrate diversity – mussel shell	Index	Diversity of macroinvertebrates on mussel shell							46.88	-	-
	Macroinvertebrate diversity – rock substrate	Index	Diversity of macroinvertebrates on rock substrate							56.77	-	55.00
	Pollution tolerance index	Index	Diatom pollution tolerance				8.47	5.13	4.27			
	Specific conductance index	Index	Specific conductance index of stream water				6.64	6.50	5.01			
	Trophic diatom index	Index	Trophic diatom index				1.00	-	4.68			

**Diversity indices**	Evenness	Index	Diatom species evenness				16.23	-	11.75			
	Richness	Index	Diatom species richness				16.23	-	12.88			
	Shannon-Weiner diversity index	Index	Shannon-Weiner diversity index of diatoms				16.23	-	11.75			
	Simpson diversity index	Index	Simpson diversity index of diatoms				16.23	-	12.88			
**Physical and water quality**	Chlorophyll a	ng/L	Stream water concentration of chlorophyll a				2.31	-	-			
	Conductivity	µS/cm	Conductivity (mussel dataset) and specific conductivity (bacterial and diatom datasets) of stream water	61.55	-	17.54	4.08	6.37	4.68	-	-	-
	Dissolved oxygen	mg/L	Stream water concentration of dissolved oxygen				12.72	6.37	6.76			
	Salinity	ppt	Stream water salinity concentration				5.20	6.27	5.01			
	Total suspended solids	mg/L	Total suspended solids in stream water	24.31	-	-	-	6.27	5.50	-	-	-

Supplemental Table S3. TDN breakpoint values (mg/L) for all parameters used in analysis. Breakpoint methods include SiZer (SZ), 2DKS, and piecewise (PW) regression. Dashes indicate that no breakpoint was detected, while empty cells indicate that no data was present for that parameter.

Type	Parameter	Unit	Description	Bacterial Dataset			Diatom Dataset			Mussel Dataset		
				SZ	2DKS	PW	SZ	2DKS	PW	SZ	2DKS	PW
Dissolved organic matter optical properties	Component 1	%	Parallel factor analysis (PARAFAC) component 1*	0.71	-	0.38				-	-	-
	Component 2	%	PARAFAC component 2*	-	-	0.33				0.78	-	1.23
	Component 3	%	PARAFAC component 3*	2.08	-	0.88				2.03	-	-
	Component 4	%	PARAFAC component 4*	0.71	0.86	0.92				2.71	0.74	1.46
	Component 5	%	PARAFAC component 5*	-	-	0.38				-	-	-
	Component 6	%	PARAFAC component 6*	1.26	0.62	1.64				2.71	0.74	1.46
	Component 7	%	PARAFAC component 7*	-	-	0.38				1.24	-	1.23
	Component 8	%	PARAFAC component 8*	-	-	0.38				-	-	-
	Component 9	%	PARAFAC component 9*	-	-	0.33				-	-	-
	β:α	Ratio	Ratio of 2 known fluorescence values*	-	-	0.66	1.67	-	2.45	-	-	0.76
	E_{280}	L/ mg·cm	Coefficient of 280nm molar absorbance*	0.87	0.89	-				-	-	-
	Fluorescence index	Index	Fluorescence index*	0.82	0.86	1.09	0.80	1.23	-	1.12	-	-
	Humification index	Index	Humification index*	0.39	-	0.31	2.67	-	2.45	-	-	-
	Redox index	Index	Redox index*	0.53	0.62	0.63	1.12	1.26	1.91	2.48	0.53	0.92
	Slope ratio	Ratio	Slope ratio*	1.98	0.63	0.67	2.33	-	2.45	-	-	-
	$SUVA_{254}$	L/ mg·cm	Coefficient of 254nm molar absorbance*	-	0.89	0.91	2.85	-	0.87	-	-	-

**Microbial biomass and activity**	α glucosidase	nmol/ L·hr	Alpha glucosidase activity	1.47	-	-						
	β glucosidase	nmol/ L·hr	Beta glucosidase activity	-	0.66	-						
	AMA	nmol/ L·hr	Aminopeptidase activity	-	-	0.52				-	-	0.76
	APA	nmol/ L·hr	Alkaline phosphatase activity	-	0.86	0.63				0.75	-	1.00
	Bacterial Growth	/day	Doubling time	1.14	0.89	-				1.92	-	-
	Bacterial production	fg C/ cell·day	Bacterial production standardized per cell	1.14	0.89	-				1.92	-	-
	Total bacterial abundance	$\times 10^9$ cells/L	Density of bacteria in stream water	-	0.56	0.56				1.80	0.56	0.87
	FungalB	mg/cm2	Fungal biomass on leaf litter as measured using ergosterol concentration							-	-	-
**Nutrient content & ratios**	$\delta^{15}N$	‰	Mussel tissue ^{15}N:^{14}N							0.25	-	-
	C:N	Ratio	Mussel tissue C:N							0.32	1.23	0.56
	C:P	Ratio	Mussel tissue C:P							-	-	-
	N:P	Ratio	Mussel tissue N:P							0.22	0.74	0.54
	O:N	Ratio	Mussel tissue O:N							-	-	-
	N excretion	µg NH_3/ hr·g/dwt	Mussel N excretion							-	-	1.26
	P excretion	µg NH_3/ hr·g/dwt	Mussel P excretion							0.75	-	0.56
	N:P excretion	Ratio	Mussel N:P excretion							0.35	-	1.26
	Periphyton C	mg/cm^2	Periphyton C conc.				-	-	-			
	Periphyton N	mg/cm^2	Periphyton N conc.				-	-	-			
	Periphyton P	mg/cm^2	Periphyton P conc.				-	-	-			
	Periphyton C:N	Ratio	Periphyton C:N ratio				-	-	-			
	Periphyton C:P	Ratio	Periphyton C:P ratio				-	-	-			
	Periphyton N:P	Ratio	Periphyton N:P ratio				-	-	-			

**Respiration & decay rates**	Brick respiration	mg/L·hr	Periphyton respiration on brick substrate							-	-	0.54
	Disk respiration	mg/L·hr	Periphyton respiration on a silicate disk substrate							-	-	0.54
	Wood respiration	mg/L·hr	Respiration of periphyton on wood substrate							1.21	2.10	0.63
	Leaf decay	mg/day	Rate of leaf-litter decay in stream water							-	-	1.26
	Wood decay	mg/day	Rate of wood decay in stream water							3.83	-	-
**Various indices**	Pigment dissimilarity index	Index	Pigment difference between mussel shell and rock substrate							3.83	0.53	-
	Macroinvertebrate dissimilarity index	Index	Macroinvertebrate difference between mussel shell and rock substrate							-	-	0.76
	Pigment diversity – mussel shell	Index	Diversity of pigments on mussel shell							-	-	0.56
	Pigment diversity – rock substrate	Index	Diversity of pigments on rock substrate							-	-	-
	Macroinvertebrate diversity – mussel shell	Index	Diversity of macroinvertebrates on mussel shell							0.18	1.49	-
	Macroinvertebrate diversity – rock substrate	Index	Diversity of macroinvertebrates on rock substrate							-	-	-
	Pollution tolerance index	Index	Diatom pollution tolerance				-	-	-			
	Specific conductance index	Index	Specific conductance index of stream water				0.57	0.87	-			
	Trophic diatom index	Index	Trophic diatom index				-	-	1.12			

**Diversity indices**	Evenness	Index	Diatom species evenness				-	-	0.76			
	Richness	Index	Diatom species richness				0.54	-	0.76			
	Shannon-Weiner diversity index	Index	Shannon-Weiner diversity index of diatoms				0.54	-	0.76			
	Simpson diversity index	Index	Simpson diversity index of diatoms				0.54	-	0.76			
**Physical and water quality**	Chlorophyll a	ng/L	Stream water concentration of chlorophyll a				-	-	-			
	Conductivity	µS/cm	Conductivity (mussel dataset) and specific conductivity (bacterial and diatom datasets) of stream water	2.19	0.60	0.63	0.86	0.91	-	-	0.56	0.92
	Dissolved oxygen	mg/L	Stream water concentration of dissolved oxygen				-	-	0.91			
	Salinity	ppt	Stream water salinity concentration				0.92	1.49	-			
	Total suspended solids	mg/L	Total suspended solids in stream water	0.53	0.89	0.63	-	-	1.62	-	-	0.60

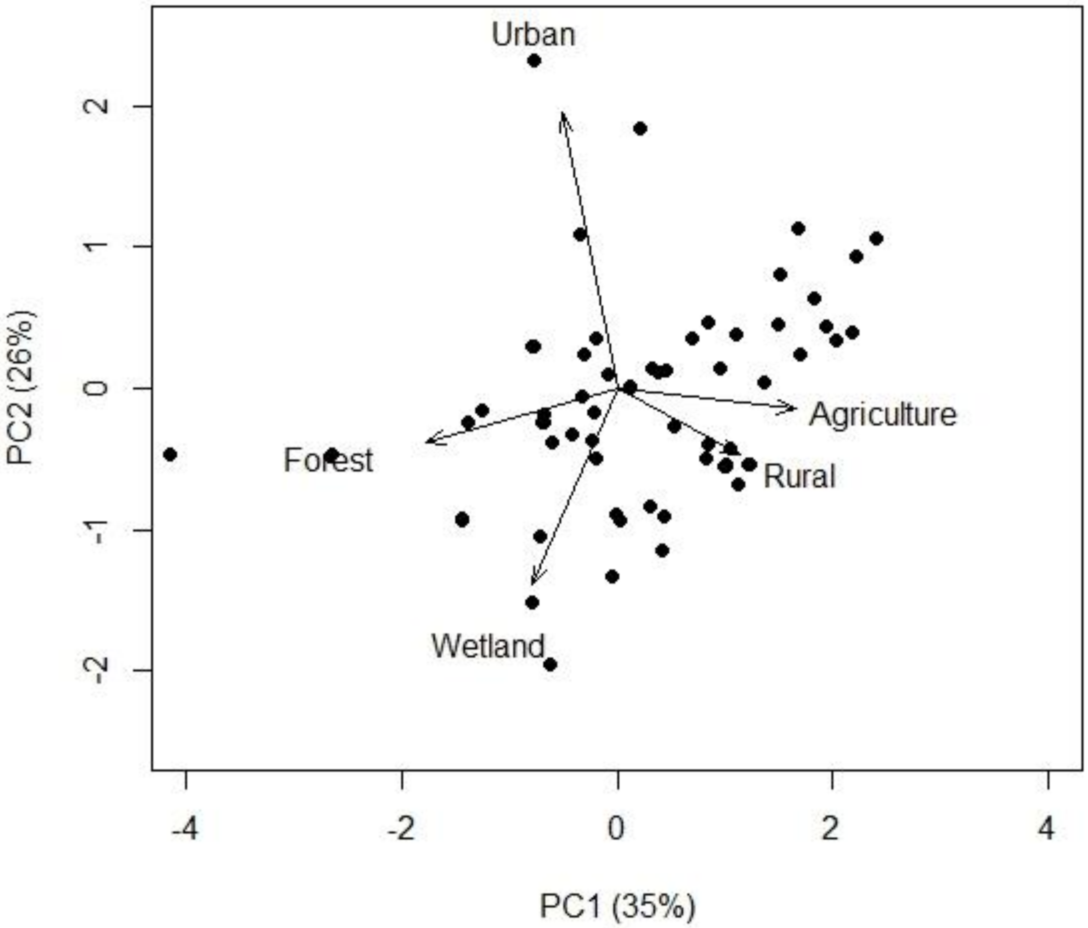

Supplemental Figure S1. Principle components analysis (PCA) of dominant land use types for sites from all three datasets. PC1 represents an agriculture-forest gradient, while PC2 represents an urban-wetland gradient.

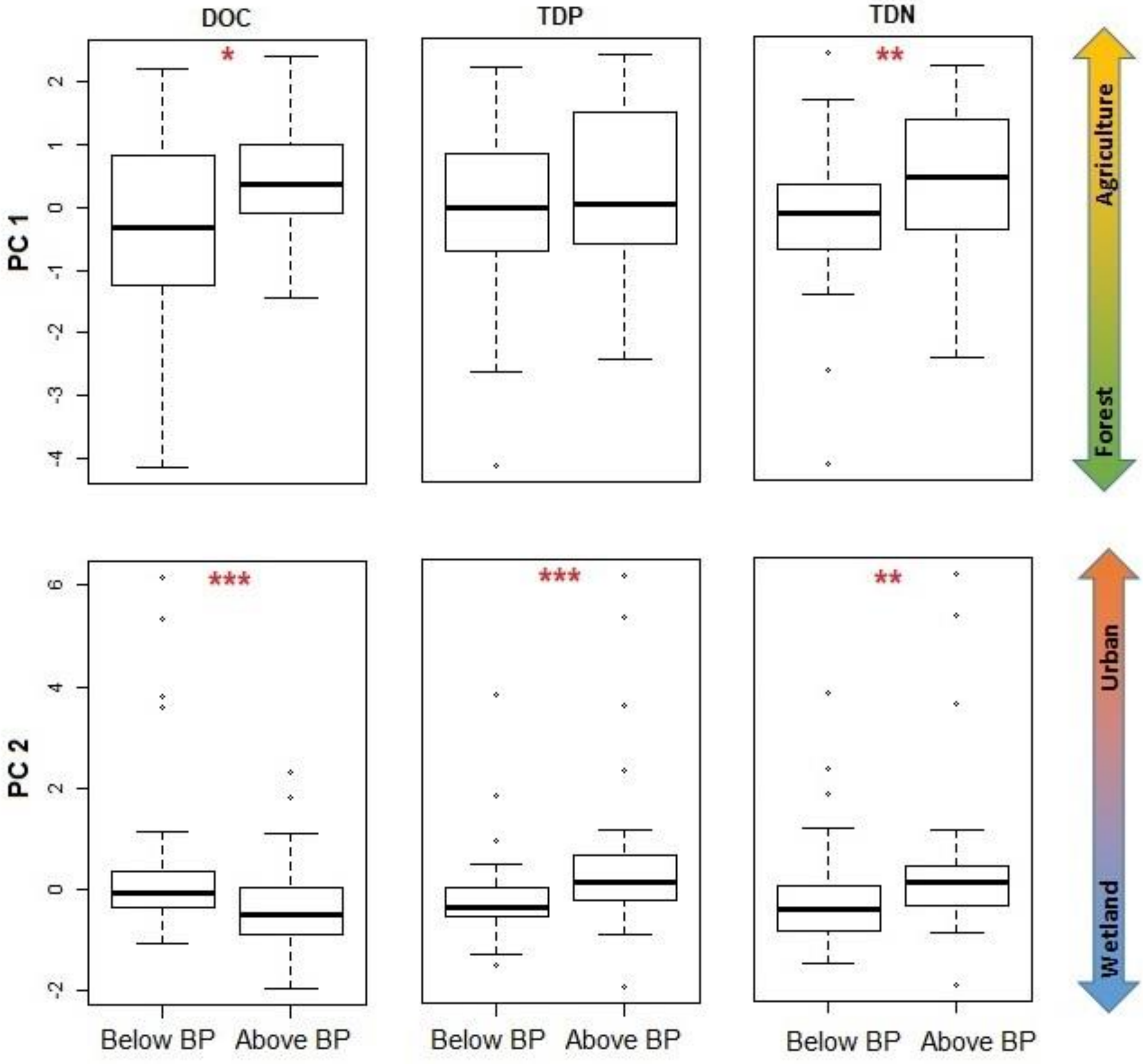

Supplemental Figure S2. Land use gradients (from principal components analysis) distributions for below- and above-breakpoint (Below BP and Above BP, respectively) for each solute using bivariate relationships in which one or more breakpoint detection method identified a breakpoint. Whiskers represent 1.5 times the interquartile range, while solid bars represent median values. Significance levels are as follows: * $p<0.05$, ** $p<0.01$, *** $p<0.001$ (Wilcoxon tests).
